# Genome-Wide Analysis of *LBD* Transcription Factor Genes in *Dendrobium* *catenatum*

**DOI:** 10.3390/ijms23042089

**Published:** 2022-02-14

**Authors:** Ru Jia, Cheng Li, Yuhua Wang, Xiangshi Qin, Lihua Meng, Xudong Sun

**Affiliations:** 1 School of Life Sciences, Key Laboratory of Yunnan for Biomass Energy and Biotechnology of Environment, Yunnan Normal University, Kunming 650500, China; jiaru19980@126.com; 2 The Germplasm Bank of Wild Species, Kunming Institute of Botany, Chinese Academy of Sciences, Kunming 650201, China; licheng@mail.kib.ac.cn (C.L.); wangyuhua@mail.kib.ac.cn (Y.W.); qinxianshi@mail.kib.ac.cn (X.Q.); 3 University of Chinese Academy of Sciences, Beijing 100049, China

**Keywords:** *Dendrobium catenatum*, *LBD* transcription factor family, phylogenetic analysis, expression profiles, phytohormone response

## Abstract

The *LATERAL ORGAN BOUNDARIES DOMAIN (LBD)* gene family comprises plant-specific transcription factors that control cell proliferation and differentiation during growth and development in many plant species. However, to date, no studies of the LBD gene family in *Dendrobium catenatum* have been reported. In this study, a genome-wide analysis of LBD genes was performed in *D. catenatum* and 24 *LBD* genes were identified. The genes were classified into two classes (I and II) based on phylogenetic relationships and motif structure. Subcellular localization analysis for DcaLBD6 and DcaLBD18 from class I and DcaLBD37 and DcaLBD41 from class II revealed that the proteins were localized in the nucleus. Transient expression analysis of DcaLBD6, DcaLBD18, DcaLBD37, and DcaLBD41 indicated that class I and class II members have opposite roles in regulating *VASCULAR-RELATED NAC-DOMAIN 7* (*VND7*) expression. *DcaLBD* genes showed diverse expression patterns in response to different phytohormone treatments. Heat maps revealed diverse patterns of *DcaLBD* gene expression in different organs. These results lay the foundation for further detailed studies of the *LBD* gene family in *D. catenatum*.

## 1. Introduction

The *LATERAL ORGAN BOUNDARIES DOMAIN* (*LBD*) transcription factors play important roles in the growth and development of many plant species. *LBD* genes are involved in the initiation, growth, metabolic regulation, and secondary growth of roots, stems, leaves, and corollas, establishment of the boundary between lateral organ and terminal meristem primordia, and have an important influence on the formation and development of aboveground and belowground organs in higher plants. In addition, *LBD* genes are involved in anthocyanin and nitrogen metabolism [1,2,3,4].

The *LBD* gene family is plant specific and is characterized by a highly conserved LATERAL ORGAN BOUNDARIES (LOB) domain. The length of the LOB domain is about 100 amino acids. The LOB domain comprises a C-block, which contains conserved cysteine residues in the CX2CX6CX3C motif required for DNA-binding activity. A Gly–Ala–Ser (GAS) block and a complete leucine zipper-like coiled-coil motif (LX6LX3LX6L) are responsible for protein dimerization [5,6]. On the basis of protein sequence analyses, LBD proteins can be classified into two groups (class I and class II). All LBD proteins contain the C-block, but LBD proteins of class I also include a leucine zipper structure, whereas class II proteins lack a complete leucine zipper structure and cannot form a spiral coil [7,8,9]. Previous studies have demonstrated that LBD proteins play important roles in plant growth, development, signal transduction, and stress response development [10,11,12,13,14,15,16].

*Dendrobium catenatum* has a broad distribution and a long history as a traditional medicinal plant in China, and has attracted considerable attention from local and international researchers on account of its high medicinal value [17]. In recent years, pharmacological research on the species has mainly focused on its antioxidant, anti-tumor, immunoregulatory, antifatigue, and diabetes-alleviating properties [18,19].

In previous studies, *LBD* transcription factors have been identified in many plant species. For example, *Malus domestica* has 58 *LBD* genes [20], *Fragaria vesca* has 35 *LBD* genes [21], *Vitis vinifera* has 40 *LBD* genes [22], *Zea mays* has 44 *LBD* genes [23], *Morus notabilis* has 31 *LBD* genes [24], *Brassica rapa* var. *rapa* has 59 *LBD* genes [25], and *Hordeum vulgare* has 24 *LBD* genes [26]. In the present study, we identified *LBD* genes in *D. catenatum*, then analyzed their structure, physicochemical properties, phylogenetic relationships, conserved motif profiles, subcellular localization, and expression patterns. Overall, this study provides valuable information for future structural and functional studies of *LBD* genes in *D. catenatum*.

## 2. Results

### 2.1. Identification of LBD Family Genes in D. catenatum

**Figure 1:**
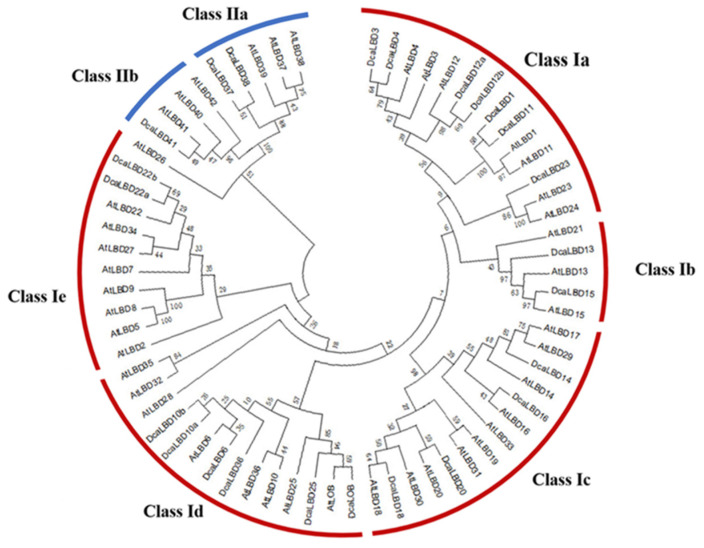
Phylogenetic tree of LBD proteins from *D. catenatum* and *Arabidopsis* was generated using the neighbor-joining (NJ) method implemented in MEGA 7.0 software. Bootstrap analysis was conducted with 1000 iterations.

To identify the LBD proteins in the genome of *D. catenatum*, a local BLAST search of the hidden Markov models (HMMs) of the SMART and Pfam databases was conducted. In total, 24 LBD genes from the complete *D. catenatum* genome were isolated. All of the deduced LBD proteins possessed a conserved LOB domain. We reconstructed a phylogenetic tree from an alignment of LBD protein sequences from *Arabidopsis thaliana* and *D. catenatum*. The *DcaLBD* genes were annotated based on the similarity of the protein sequences to those of *A. thaliana* (Figure 1).

On the basis of the phylogenetic analysis, the LBD proteins of *D. catenatum* were resolved into two monophyletic groups (classes I and II), of which 21 LBD proteins belonged to class I and three LBD proteins belonged to class II. Sequence analysis revealed that AtLBD1, 3, 4, 6, 10, 11, 12, 13, 14, 15, 16, 18, 20, 22, 23, 25, 36, 37, 38, 41, and LOB have orthologs in *D. catenatum* of which DcaLBD10, 12, and 22 comprised multiple orthologs. 

The length of the DcaLBD proteins ranged from 148 to 307 aa, the molecular weight ranged from 17.06 to 34.33 KDa, the theoretical isoelectric point value ranged from 4.61 to 9.33, and the calculated grand average of hydrophobicity value ranged from −0.529 to 0.114. Most of the proteins were hydrophobic with the exception of DcaLBD4 and DcaLBD16 (Table 1).

### 2.2. Motif Analysis and Gene Structure

**Figure 2:**
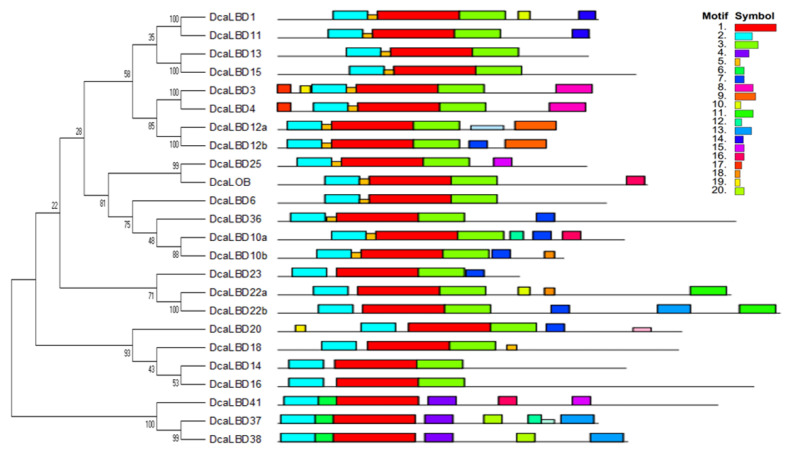
Genomic structure and motif composition of *D. catenatum* LBDs, phylogenetic tree of *D. catenatum* LBD proteins. The conserved motifs in *D. catenatum* LBD proteins were identified using MEME, each motif is shown in a specific color.

To further explore the diversity of *LBD* genes in *D. catenatum*, the conserved motifs of the DcaLBD proteins were analyzed. A neighbor-joining (NJ) tree was constructed based on a multiple alignment of the DcaLBD protein sequences. The MEME online tool was used to predict the conserved motif composition of the DcaLBD proteins. The number of motifs ranged from three to seven. A LOB domain, motif 2, was detected in all DcaLBDs. In addition, motif 3 was detected in all class I members, indicating that the class I proteins have a specific structure (Figure 2).

**Figure 3:**
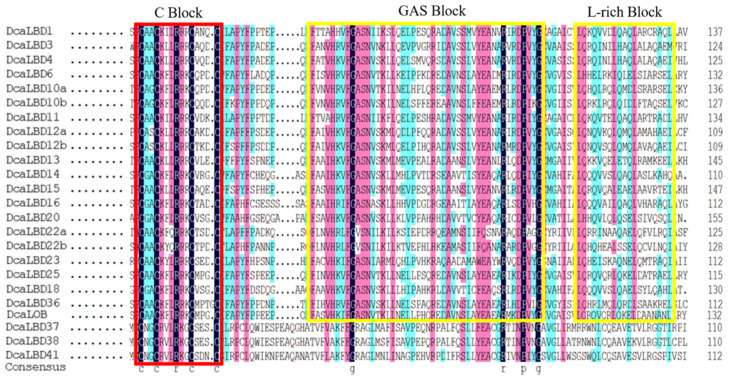
LBD-domain alignment and corresponding logo of DcaLBDs. The conserved C–block in all DcaLBDs protein is indicated by a red box. The GAS block and L-rich block in class II of DcaLBDs protein are indicated by yellow boxes.

On this basis, we further analyzed the DcaLBD protein sequences using the DNAMAN tool. The multiple sequence alignment indicated that a sequence comprising more than 100 amino acids was conserved in all DcaLBDs (Figure 3). For the class I proteins, a string consisting of a C-block, GAS-block, and L-rich block was detected. The C-block in DcaLBDs can be summarized as CX_2_CX_6_CX_3_C. The GAS-block began with a FX_2_V/AH motif and ended with a DPV/IYG motif. All class II DcaLBDs contained the conserved C-block similar to class I proteins and lacked the GAS-block and Leu-zipper-like domain.

### 2.3. Subcellular Localization

**Figure 4:**
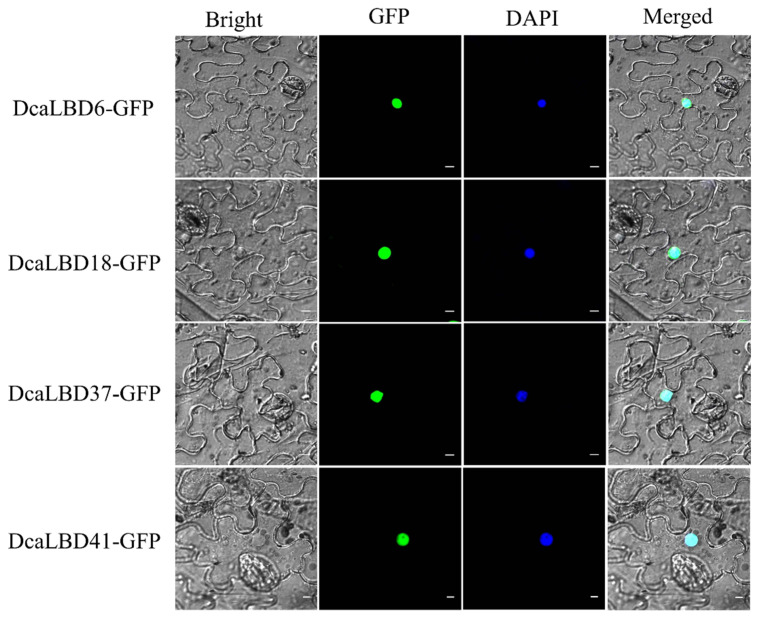
Subcellular localization of 35S:DcaLBD-GFP in *Nicotiana benthamiana* leaves. DcaLBD6-GFP, DcaLBD18-GFP, DcaLBD37-GFP and DcaLBD41-GFP were localized in the nucleus. Bar = 10 μm.

We predicted that the *LBD* gene family were transcription factors localized in the nucleus to play roles in regulating plant growth and development. To support this hypothesis, we conducted a subcellular localization analysis for DcaLBD6 and DcaLBD18 from class I and DcaLBD37 and DcaLBD41 from class II. The respective *DcaLBD* gene was fused with the green fluorescent protein (GFP) gene and co-injected into *Nicotiana benthamiana* leaves. The transfected plants were cultured in a greenhouse for 3 days. The fluorescence in the injected leaves was observed under a microscope (Olympus FV1000, Tokyo, Japan) after 4′,6-diamidino-2-phenylindole (DAPI) injection. Fluorescent signal from the DcaLBD–GFP protein was detected in the nucleus, consistent with the proteins functioning as transcription factors (Figure 4).

### 2.4. Class I and II LBDs Regulate VND7 Expression

**Figure 5:**
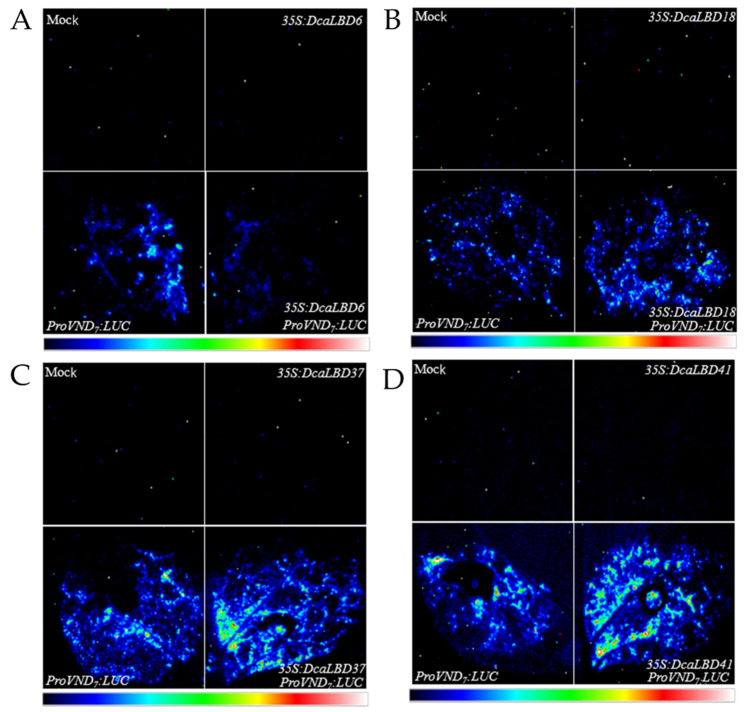
Transient expression analysis of DcaLBD6, DcaLBD18, DcaLBD37 and DcaLBD41 activities. (**A**) *VND7* was repressed by DcaLBD6. (**B**) *VND7* was activated slightly by DcaLBD18. (**C**) *VND7* was activated by DcaLBD37. (**D**) *VND7* was activated by DcaLBD41.

Previous studies have shown that members of the *LBD* family have a positive feedback-regulatory effect on the master regulator VASCULAR-RELATED NAC-DOMAIN 7 (VND7) [27]. Therefore, we speculated that the DcaLBD family members may also regulate the expression of *VND7*. To test this hypothesis, we co-injected the *ProAtVND7:LUC* promoter with class I (*35S:DcaLBD6* and *35S:DcaLBD18*) or class II (*35S:DcaLBD37* and *35S:DcaLBD41*) gene constructs (Figure 5). When the *ProAtVND7:LUC* reporter plasmid was co-injected with the *35S:DcaLBD37* or *35S:DcaLBD41* effector plasmid, intense luciferase (LUC) fluorescence signal was detected. These results indicated that *DcaLBD37* and *DcaLBD41* directly activated expression of *AtVND7*. In contrast, when the *ProAtVND7:LUC* reporter plasmid was co-injected with the *35S:DcaLBD6* effector plasmid, faint LUC fluorescence signal was detected, but when injected without the effector plasmid the LUC fluorescence signal was weaker. Thus, *DcaLBD6* restrained expression of *AtVND7*. Co-injection of the *ProAtVND7:LUC* reporter plasmid with the *35S:DcaLBD18* effector plasmid resulted in LUC fluorescence signal similar to that of the *ProAtVND7:LUC.* These results are consistent with previous studies of LBD family members in suggesting that DcaLBD family members exhibit different functions or even antagonistic roles [28].

### 2.5. Expression Profiles of LBD Genes in D. catenatum

To investigate the function of *LBD* genes in *D. catenatum*, we used transcriptomic data to determine changes in the expression of the *DcaLBD* family genes under different phytohormone treatments.

**Figure 6:**
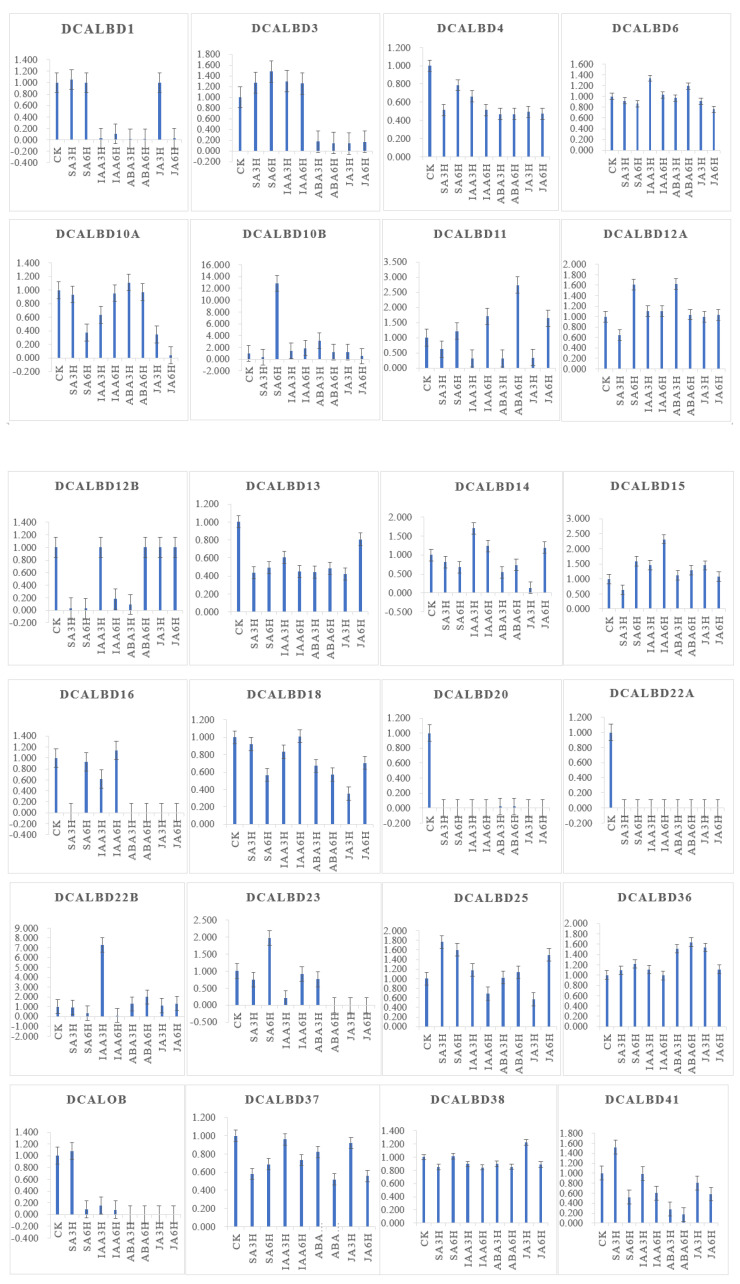
Expression analysis of *DcaLBD* genes after treatment with ABA, IAA, JA and SA. ABA: abscisic acid, IAA: indole-3-acetic acid, JA: jasmonic acid, SA: salicylic acid. ck: control, 3 h: treatment with phytohormone after 3 h, 6 h: treatment with phytohormone after 6 h.

*DcaLBD13* showed the highest relative expression level without treatment (Figure 6). The expression level of *DcaLBD13* decreased to varying degrees under the various phytohormone treatments. After treatment with indole-3-acetic acid (IAA) for 3 h, *DcaLBD6* showed the highest expression level. After treatment with IAA for 6 h, the expression level of *DcaLBD18* was the highest. Among class II genes, *DcaLBD38* showed the highest expression level without treatment. After treatment with jasmonic acid (JA) for 3 h, the expression level of *DcaLBD38* was increased. The expression levels of *DcaLBD37* were increased after phytohormone treatment. *DcaLBD41* showed the highest expression level after treatment with salicylic acid (SA) for 3 h.

**Figure 7:**
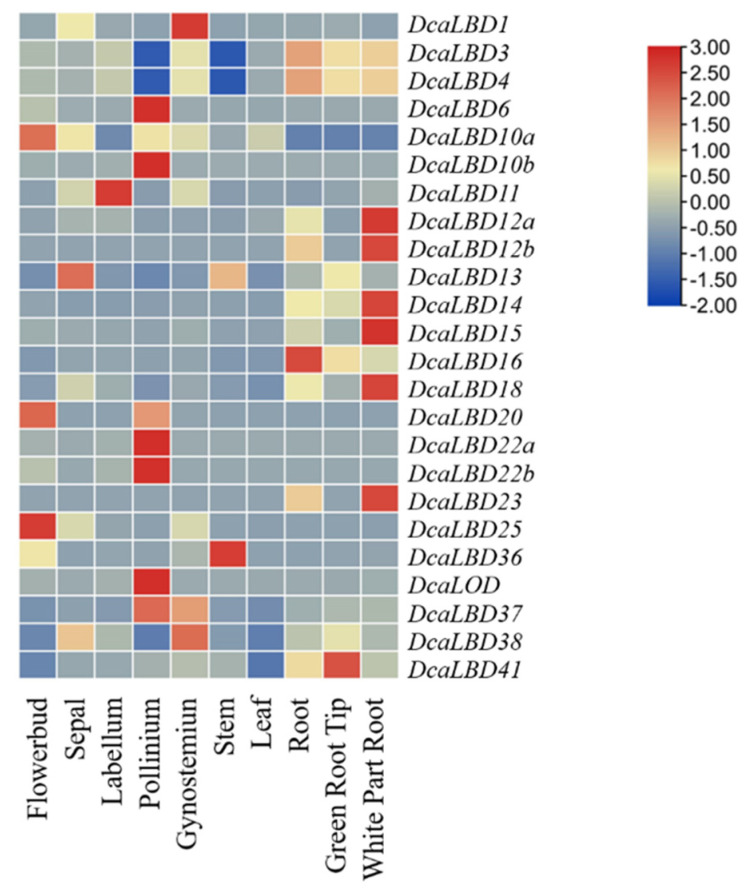
Expression analysis of *DcaLBD* genes in different organs.

Heat maps were generated to indicate the relative expression of the *DcaLBD* family members in different organs (Figure 7). Among class I genes, *DcaLBD22b* showed the highest expression level in the pollinium. *DcaLBD6* was more highly expressed in the pollinium than in other organs. The expression of *DcaLBD18* was higher in the white portion of the root than in other organs. Among class II genes, *DcaLBD38* showed the highest expression level in the gynostemium. The expression level of *DcaLBD37* and *DcaLBD41* in the pollinium and green root tip were higher than that in other organs.

## 3. Discussion

As plant-specific transcription factors, the LBD family has been studied extensively in plants. Plants have evolved from low to high, and the number of *LBD* family genes shows a trend of expanding from scratch. The number of *LBD*s in different species of plants is different, and the genome size of lower plants is relatively small and the structure is relatively simple, so the number of *LBD* gene family members is relatively small. Previous studies have shown that the *LBD* gene family members are absent in *Chlamydomonas reinhardtii* and *Volvox carteri*, while 26 *LBD* gene family members are present in *physcomitrella patens* in bryophytes, 15 *LBD* gene family members are present in *selaginella moellendorrffii* in ferns, and more than 20 *LBD* gene family members are present in most angiosperms [29]. For example, *A. thaliana* has 43 *LBD* genes and *D. catenatum has 24 LBD genes*. In previous studies, there are 4, 1, 1 *LBD* gene family members were found from *Nitella mirabilis*, *Coleochaete Orbicularis* and *Spirogyra pratensis*. The fact that there are several *LBD* gene family members in Charales suggests that LBD proteins were already exist before algae evolved towards terrestrial plants. Charales is the closet ancestor of higher embryo plants [30]. After two rounds of genetic replication, these primitive gene lineages formed the ancestral genes of partial gymnosperms and angiosperms. In the process of terrestrial plant evolution, the overall replication and discrete replication of the whole genome greatly increased the number of *LBD* genes in angiosperms, and the frequent changes in protein replacement and expression patterns promoted the diversification of *LBD* genes, so the *LBD* transcription factor family differentiation produced a variety of biologically functional proteins [31]. These results show that *LBD* gene expands the number of genes and diversifies its functions through a complex gene replication process in the process of family evolution. The *LBD* gene family members in *A. thaliana* and *D. catenatum* as angiosperms are more numerous and functionally more complete.

In *Arabidopsis*, AtLBDs are classified into two classes, of which members of class I have a leucine zipper structure, whereas class II members lack a complete leucine zipper structure [7]. Thus, the functions of class I and II *LBD* genes differ. Class I members are associated with organ development and stress resistance [10,11,12,13]; for example, *LBD15* is associated with drought resistance and regulates the expression of *VND7* [28]. In contrast, the functions of class II members are involved in metabolism [2]. Therefore, the *LBD* gene family members exhibit functional diversity. In the present study, DcaLBDs were classifiable into classes I and II based on the characteristic motifs. We also observed that *DcaLBD* genes regulated the expression of *VND7*, which is a NAC-domain transcription factor that regulates xylem formation [32]. Previous studies on *Arabidopsis* have shown that *AtLBD6* has an important regulatory effect on the symmetrical development of leaves and the normal development of vascular system [6]. Overexpression of *TcLBD6* can lead to a significant reduction in development of lateral roots. *AtLBD16* and *AtLBD18* jointly regulate the development of *Arabidopsis* lateral roots under the upstream regulation of *AtARF* [33], whereas *AtLBD6* does not show this function. These results suggest that *LBD6*-related genes are typically pleiotropic genes, and that physiological effects in plants may differ. *Eucalyptus grandis LBD* genes differ in degree of influence on secondary growth; overexpression of *EgLBD37* results in a significant increase in secondary xylem development, whereas overexpression of *EgLBD29* leads to a significant increase in phloem fiber development [34]. These results suggest that *DcaLBD37* and *DcaLBD41* might be involved in xylem formation via regulation of the expression of *VND7*. These findings suggest a potential mechanism for the function of *DcaLBD* genes. However, the roles of *DcaLBD* genes and their mechanism of action need to be further studied in the future.

Plant hormones affect the expression of genes through diverse mechanisms [35,36]. The expression patterns of *DcaLBD* genes differed among plant organs, indicating that different *DcaLBD* genes were involved in different organ development processes. It further suggests that the functions of *LBD* gene family members are diverse. The positive feedback-regulatory effect on *LBD* gene and NAC (NAM/ATAF/CUC) protein regulates growth of *A. thaliana* by controlling the differentiation of xylem cells. *LBD16*, *LBD18*, and *LBD29* are also involved in regulating the formation of lateral roots in *Oryza sativa* and *Zea mays* [7,23]. In this study, the heatmap of expression analysis of *DcaLBD* genes in different organs (Figure 7) also showed that the expression of *DcaLBD16* and *DcaLBD18* in the root was higher than that of other organs. These results suggest that the *LBD* gene family plays an important role in secondary growth processes that affect the quality of plant development. Under the influence of IAA [36], the expression of *DcaLBD15*, *DcaLBD16* and *DcaLBD18* increased, which was in line with the expected results. *AtLBD10* and *AtLBD22* were involved in pollen formation, and the expression of *DcaLBD10* and *DcaLBD22* in pollinium was significantly higher than in other parts of the Figure 7, and the results were also in line with expectations [14]. The function of MdLBD11 in *Malus domestica* is similarly to AtLBD11 [20], where the expression of *LBD11* may affect plant phenotype includes abnormal traits such as leaf curling upwards, delayed flowering, flowering downwards, and siliques. In this study, the expression data of *DcaLBD11* in labellum is also significantly higher than that of other organs, and it can be inferred that the function of *DcaLBD11* may be similar to that of *A. thaliana* and *Malus domestica*.

In summary, 24 *LBD* genes were identified in *D. catenatum* and classified into classes I and II. Expression analysis suggested that DcaLBD transcription factors perform various functions. The present results provide an important foundation for further study of the functions of *DcaLBD* genes. At present, the research on the *LBD* transcription factors mainly focuses on the discovery of new members and the study of biological functions, but the study of its origin, evolution and the analysis of the characteristics of each subclass needs to be deepened [31].

## 4. Materials and Methods

### 4.1. Plant Material and Phytohormone Treatments

Plants of *Dendrobium catenatum* were grown in a sand:soil mixture (1:3, *w*/*w*). The plants were cultivated in a greenhouse maintained at 23 °C under relative humidity of 65–75% and a 12 h (daytime, 08:00–20:00) photoperiod (50 μmolm^−^^2^ s^−^^1^). Treatments for *D. catenatum* followed the methods of Zhang [37]. Non-treated plants were used as the control. The collected samples were immediately frozen in liquid nitrogen and stored at −80 °C until further use.

### 4.2. Identification of LBD Family Genes in D. catenatum

To identify the members of the LBD gene family in *D. catenatum*, three different approaches were performed. First, proteins data for the whole genome sequence of *D. catenatum* were downloaded from OrchidBase (http://orchidbase.itps.ncku.edu.tw/est/Dendrobium). All known *Arabidopsis LBD* gene and protein sequences were downloaded from TAIR 12.0 (www.arabidopsis.org) [38]. Local BLASTP and TBLASTN (Basic Local Alignment Search Tool, http://blast.ncbi.nlm.nih.gov) tools [39], available from the National Center for Biotechnology Information (NCBI) website, were used to search the genome sequence of *D. catenatum* with the known AtLBD sequences as the query. These searches identified the initial candidate genes containing putative LOB domains in *D. catenatum*. Second, the HMM profile (accession number PF03195) from the Pfam database (http://pfam.sanger.ac.uk) [40] was applied to confirm the presence of the conserved LOB domain in each candidate LOB motif. Finally, DNAMAN 7.0 software was used for multiple sequence alignment and amino acid sequence analysis of *D. catenatum* LBD proteins and the LOB domain motif (CX2CX6CX3C). The sequences of the identified *LBD* genes were confirmed using the conserved domain search tool. The retrieved sequences of candidate *D. catenatum LBD* genes that lacked a LOB domain were discarded.

### 4.3. Gene Structure and Conserved Motif Analysis

The conserved motifs of DcaLBD proteins were analyzed using the Multiple Em for Motif Elucidation (MEME) website (http://meme-suite.org/index.html) [41]. The search parameters were set as follows: the optimal motif order width was set from 6 to 50, the maximum number of motifs identified was 20 motifs, and all other parameters were set to the default value. In addition, the sequence was used is appeared at least once. 

### 4.4. Phylogenetic Analysis and Physicochemical Properties

The LOB domain nucleotide sequences of *DcaLBD* and *AtLBD* genes were used in the phylogenetic analysis. We used MEGA 7.0 software [42] to generate a multiple sequence alignment and perform a neighbor-joining (NJ) analysis with 1000 bootstrap replicates. Using Expasy Protparam tool (ExPASy–ProtParam tool) [43] to analyze the identified DcaLBDs, including their CDS lengths, the protein sizes, protein molecular weight (MW), isoelectric points (pI), and grand average of hydrophilicity (GRAVITY) of DcaLBD genes.

### 4.5. Subcellular Localization 

The CDS of *DcaLBD6*, *18*, *37*, and *41* was amplified using Phanta^®^ Max Super-Fidelity DNA Polymerase (Vazyme Biotech Co., Ltd., Nanjing, China) with relevant primers (Table S1). The ClonExpress^®^ II One Step Cloning Kit (Vazyme Biotech Co., Ltd.) was used to insert the CDS into the pRI101-GFP vector. The *35S:GFP-DcaLBD* constructs were transferred into *Agrobacterium tumefaciens* strain EHA105 using electroporation and then injected into *Nicotiana benthamiana* leaves. The transfected plants were cultivated for 3 days in a greenhouse. The injected leaf blade after 4′,6-diamidino-2-phenylindole (DAPI) injection was excised and observed with a laser confocal microscope [25,33].

### 4.6. Transient Expression in Nicotiana benthamiana Leaves

Transactivation activity detection assays were performed using *35S:GFP-DcaLBD6*, *18*, *37*, and *41* as the effector and *ProVND7*:LUC as the reporter. The *35S:GFP-DcaLBD6*, *18*, *37*, and *41* constructs were co-injected with *ProAtVND7:LUC* into *N. benthamiana* leaves with a syringe. The transfected plants were cultured in a greenhouse for 3 days. Before observation, 1 mM luciferin solution was sprayed evenly on the surface of the injected *N. benthamiana* leaves. The leaf blades were then incubated at a low temperature in the dark for 3 min. Luciferase luminescence was captured using a Tanon-5200 Chemiluminescent Imaging System with a low-light cooled CCD camera [44].

### 4.7. Expression Analysis

Transcriptomic data from phytohormone-treated *D. catenatum* were downloaded from the Biodiversity Data Center (iflora.cn). The data is collated and a histogram is made by Excel, with ck as 1. The expression level of the *DcaLBD* genes in different organs (flower bud, sepal, labellum, pollinium, gynostemium, stem, leaf, root, green root tip, and white portion of the root) of *D. catenatum* were retrieved from OrchidBase (http://orchidbase.itps.ncku.edu.tw/est/). A heat map for tissue-specific expression of the *DcaLBD* genes was constructed using Tbtools [45]. All data were adjusted using normalize genes. Hierarchical clustering was performed using the default parameters [46].

## 5. Conclusions

In this study, 24 LBD transcription factors were identified in the genome of *D. catenatum* and a comprehensive analysis of this gene family was performed. The genes were classified into classes I and II and showed functional diversity; even members within the same class showed opposite functions. The expression patterns of *DcaLBD* genes showed diverse responses to phytohormone treatments and differential expression patterns in different organs. This study provides a basis for selection of candidate genes to elucidate the functional roles of *DcaLBD* in the growth and development of *D. catenatum*.

## Figures and Tables

**Table 1:** Identification and characteristics of *LBD* genes in *D. catenatum*.

Gene Name	Accession Number	CDS Length (bp)	Protein Size (aa)	MW (kD)	PI	GRAVY
DcaLBD1	XP_020695176.1	591	196	21.6	5.77	−0.172
DcaLBD3	XP_020704279.1	579	192	20.84	8.98	−0.049
DcaLBD4	XP_020673109.1	567	188	20.27	6.93	0.114
DcaLBD6	XP_020676861.1	606	201	21.37	8.56	−0.115
DcaLBD10a	XP_020702209.1	639	212	22.85	7.64	−0.323
DcaLBD10b	XP_020700451.1	528	175	19.53	9.28	−0.225
DcaLBD11	XP_020701018.1	576	191	20.79	6.40	−0.020
DcaLBD12a	XP_020682070.1	513	170	18.84	6.07	−0.152
DcaLBD12b	XP_020701020.1	495	164	18.36	6.94	−0.291
DcaLBD13	XP_020682962.1	573	190	21.06	8.27	−0.247
DcaLBD14	XP_020677496.1	642	213	23.32	6.11	−0.139
DcaLBD15	XP_020682881.2	660	219	23.82	8.85	−0.235
DcaLBD16	XP_020701180.1	876	291	31.13	9.33	0.047
DcaLBD18	XP_020699320.1	738	245	25.76	8.26	−0.212
DcaLBD20	XP_020693551.1	744	247	26.93	6.35	−0.300
DcaLBD22a	XP_020686925.1	834	277	31.15	4.61	−0.465
DcaLBD22b	XP_020682680.1	924	307	34.33	5.15	−0.443
DcaLBD23	XP_028557256.1	447	148	17.06	8.70	−0.418
DcaLBD25	XP_028549413.1	681	226	24.66	5.96	−0.345
DcaLBD36	XP_020698423.1	843	280	31.14	6.81	−0.529
DcaLOB	XP_020678334.1	570	189	21.32	8.26	−0.108
DcaLBD37	XP_028551170.1	591	196	21.76	6.17	−0.297
DcaLBD38	XP_020688010.1	645	214	23.37	8.74	−0.257
DcaLBD41	XP_020703175.1	810	269	29.17	7.56	−0.442

## Data Availability

Not applicable.

## Supplementary Materials

The following are available online at https://www.mdpi.com/article/10.3390/ijms23042089/s1.
